# Triangular Fibrocartilage Characterization with Ultrashort Echo Time-T2* MRI: Insights from a Healthy Cohort

**DOI:** 10.3390/life15071117

**Published:** 2025-07-17

**Authors:** Sana Boudabbous, Hicham Bouredoucen, David Ferreira Branco, Stefan Sommer, Tom Hilbert, Pierre-Alexandre Poletti, Rares Salomir, Bénédicte Marie Anne Delattre

**Affiliations:** 1 Division of Radiology, Diagnostic Department, Geneva University Hospital, 1211 Geneva, Switzerlandbenedicte.delattre@hug.ch (B.M.A.D.); 2 Image Guided Interventions Laboratory (GR-949), Faculty of Medicine, University of Geneva, 1211 Geneva, Switzerland; 3 Swiss Center for Musculoskeletal Imaging (SCMI), Balgrist Campus, 8008 Zurich, Switzerland; 4 Advanced Clinical Imaging Technology (ACIT), Siemens Healthineers International AG, 1015 Lausanne, Switzerland; 5 Department of Radiology, Lausanne University Hospital and University of Lausanne, 1011 Lausanne, Switzerland; 6 LTS5, Ecole Polytechnique Fédérale de Lausanne (EPFL), 1015 Lausanne, Switzerland

**Keywords:** TFCC, age, ulnar variance, quantitative MRI imaging, UTE-T2*, degeneration

## Abstract

The objective of this study is to measure T2* relaxation time in the triangular fibrocartilage (TFC) disc in asymptomatic volunteers and evaluate its variation with factors such as age, hand dominance, sex, and ulnar variance, using a dedicated MRI sequence. The MRI protocol included anatomical sequences as well as a 3D ultra-short echo time (UTE)-T2* mapping sequence. A linear regression model was used to assess the potential influence of age, sex, and hand dominance on T2* values measured in the TFC disc and to evaluate the correlation between T2* values and ulnar variance. T2* relaxation time was positively correlated with age. The higher T2* relaxation times may reflect early degeneration of the fibrocartilage microstructure, which is associated with both biomechanical factors and the aging process. However, T2* was not significantly influenced by sex or hand dominance, nor was it correlated with ulnar variance (this later being limited by the fact that none of our subject had positive ulnar variance). In conclusion, UTE-T2* is a promising MRI technique showing positive correlation with age in the TFC of healthy subjects. These findings are a first step to establish normative T2* values and will help interpreting deviations observed in patient with suspected pathology in future studies.

## 1. Introduction

The triangular fibrocartilage complex (TFCC) is composed of the central fibrocartilaginous disc associated with ligaments, the extensor carpi ulnaris tendon and the meniscus homolog [1]. A positive ulnar variance has been associated with TFCC degeneration, predisposing individuals to ulnocarpal impingement [2]. Degenerative changes in the TFCC (resulting from chronic, progressive tissue deterioration due to aging or overuse) often include an increase in apoptotic cells, as well as loss of elasticity and collagen fibers [3,4] and central disc degeneration becomes more common with advancing age [2].

TFCC injuries can be classified according to the Palmer and Werner classification [5]. Degenerative tears, typically classified as type 2, produce vague distal radioulnar pain associated with daily activities and are commonly related to ulnocarpal impaction [5]. They appear as central disc thinning, progressing to disc perforation, and in advanced stages, ulnocarpal arthritis [6]. Degenerative tears must be distinguished from traumatic central disc perforations (classified as 1A), which results from acute structural damage due to injury, however this distinction can be challenging [7]. Moreover, debridement and partial excision of the central disc are recommended for traumatic tears but are more controversial in degenerative cases [8,9]. A recent systematic review revealed a high prevalence of TFCC tears in asymptomatic wrists [10], suggesting that this incidental findings should not be mistaken as the primary cause of ulnar-sided pain, which could otherwise lead to unnecessary arthroscopic intervention [11,12].

Magnetic Resonance Imaging (MRI) is a non-invasive medical imaging technique that uses strong magnetic fields and radiofrequency waves to create detailed images of the body’s internal structures. In musculoskeletal imaging, MRI is particularly useful for visualizing soft tissues such as muscles, ligaments, cartilage, and bone marrow. It provides crucial information for diagnosing injuries, inflammation, and degenerative conditions without using ionizing radiation. MRI also enables quantitative imaging, such as T1, T2, or T2* mapping using dedicated techniques, offering unique insights into tissue microstructure and environment.

However, quantitative assessment of the TFCC is rarely reported in the literature. Only one study has investigated the influence of wrist position on UTE-T2* values of the triangular fibrocartilage. This study, found that T2* was lower in pronation compared to neutral and wrist flexion positions, likely due to differences in the loading of the articulation in these positions [13]. In contrast, the use of UTE-T2* imaging has been widely studied in other fibrocartilaginous structures, such as the meniscus, spinal discs, Achilles tendon, and temporomandibular joints [14,15,16,17,18,19,20,21]. T2* has shown a high correlation with the severity of degeneration [18,22]. An application of T2* mapping would be to facilitate the differentiation between traumatic and degenerative TFC defects in routine clinical practice. However, the first step is to establish baseline T2* characteristics in a healthy population. Establishing normative T2 values in asymptomatic volunteers is a critical first step toward identifying pathological deviations, thereby enabling the differentiation between traumatic and degenerative TFCC lesions in clinical populations.

Therefore, the aim of this study was to measure UTE-T2* in volunteers and evaluate its variation with factors such as age, hand dominance, sex, and ulnar variance.

## 2. Materials and Methods

### 2.1. Population

Ten asymptomatic volunteers participated in this pilot study, and both of their hands were scanned. All volunteers were non-manual workers with no history of wrist trauma, specifically no prior injury or surgery involving the triangular fibrocartilage complex (TFCC) to reduce confounding factors and variability, with the aim of establishing preliminary normative values. Research was conducted in accordance with the Declaration of Helsinki. All volunteers provided written informed consent for the use of their data, and ethical approval for image analysis was obtained from the local ethics committee (CE: 2017-00922).

### 2.2. MRI Protocol

Examinations were conducted using a 3T MRI scanner (MAGNETOM Vida, Siemens Healthineers AG, Forchheim, Germany) equipped with a dedicated 16-channel hand-wrist coil. Subjects were positioned prone with the forearm extended above the head and the wrist in a prone position, referred to as the “superman” position. The imaging protocol consisted of six sequences, including a research application 3D UTE-T2* mapping sequence. This sequence is a gradient echo-based radial sequence. The imaging parameters are summarized in Table 1. T2* maps were automatically generated on the scanner inline performing a mono-exponential fit. The total scan duration per side was 25 min.

### 2.3. UTE-T2* Measurement in the TFC Disc

Manual segmentation of the triangular fibrocartilage (TFC) disc was performed on the UTE-T2* maps using Osirix software v14.1.0 [23] (Bernex, Switzerland) by a physicist. To avoid including ligaments and other structures within the TFC complex, segmentation was guided by simultaneously displaying the 3D proton density (PD) sequence with fat suppression alongside the UTE-T2* map. Regions of interest were drawn on all slices where the TFC disc was visible, and the mean T2* value was calculated for the resulting volume of interest. Segmentations were reviewed by a senior radiologist with 17 years of experience.

### 2.4. Ulnar Variance Measurement

Ulnar variances were measured in all subjects by a senior radiologist using the T1 coronal sequence. It describes the relative length of the ulna compared to the radius at the wrist joint (positive: the ulna is longer than the radius, negative: the ulna is shorter than the radius, neutral: ulna and radius are the same length). To distinguish between neutral and positive ulnar variance, cutoff was defined as 1 mm. Although ulnar variance is highly position-sensitive and typically assessed with conventional radiography (the gold standard), studies have demonstrated a strong correlation between MRI and radiographic measurements, indicating that MRI is a reliable method for assessing ulnar variance [24,25].

### 2.5. Statistical Analyses

Statistical analyses were conducted using R software (R Core Team (2024)). R: A Language and Environment for Statistical Computing. R Foundation for Statistical Computing, Vienna, Austria. URL https://www.R-project.org/ (accessed on 15 June 2024). Mean T2* values in the TFC disc were correlated with subject’s age, sex, and dominant hand. A linear regression model was used to evaluate the potential influence of age, sex, and dominant hand on T2* values. In the initial (simple) model, T2* was specified as the dependent variable, and age, sex, and dominant hand were included as independent variables. To further explore potential interaction effects, a more complex model was constructed by adding interaction terms between age and sex, as well as between age and dominant hand, to assess whether the relationship between age and T2* varied by sex or by hand dominance.

An ANOVA test was used to assess the statistical difference between the simple and complex models. Additionally, a separate linear regression model was applied to evaluate the correlation between T2* values and ulnar variance. A significance level of *p* ≤ 0.05 was used for all analyses.

## 3. Results

**Figure 1:**
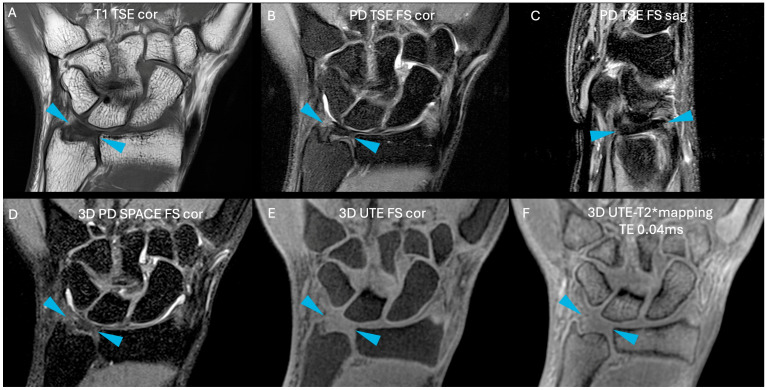
Example of all sequences acquired for the right side of a volunteer. (**A**) T1 TSE in coronal orientation; (**B**) PD TSE with fat suppression in coronal orientation; (**C**) PD TSE with fat suppression in sagittal orientation; (**D**) 3D PD SPACE with fat suppression in coronal orientation; (**E**) 3D UTE with fat suppression in coronal orientation; (**F**) and the first echo of the 3D UTE-T2* mapping sequence in coronal orientation (TE = 0.04 ms). Cortical bone appears hypointense on all sequences. Fluids appear hyperintense on T2- and PD-weighted images and hypointense on T1-weighted image. Fat suppression helps distinguish fluids from fat, as the latter is also hyperintense. Blue arrows indicate TFC.

**Figure 2:**
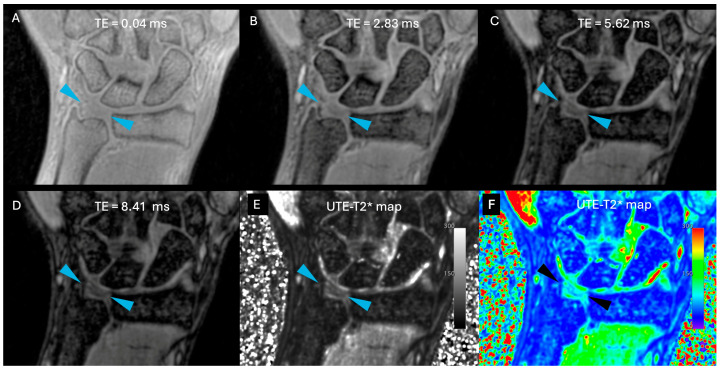
(**A**–**D**): the same slice of the four different TEs used in the 3D UTE-T2* mapping sequence; (**E**) the resulting UTE-T2* map in grayscale, and (**F**) the UTE-T2* map in color (using the “RAINBOW” colormap in Osirix). The scaling is set from 0 to 300 a.u., corresponding to 0 to 30 ms. Increasing TE will cause tissues with short T2 relaxation times (e.g., fat) to appear hypointense, while tissues with long T2 relaxation times (e.g., muscle) will appear hyperintense. Blue arrows indicate TFC.

**Figure 3:**
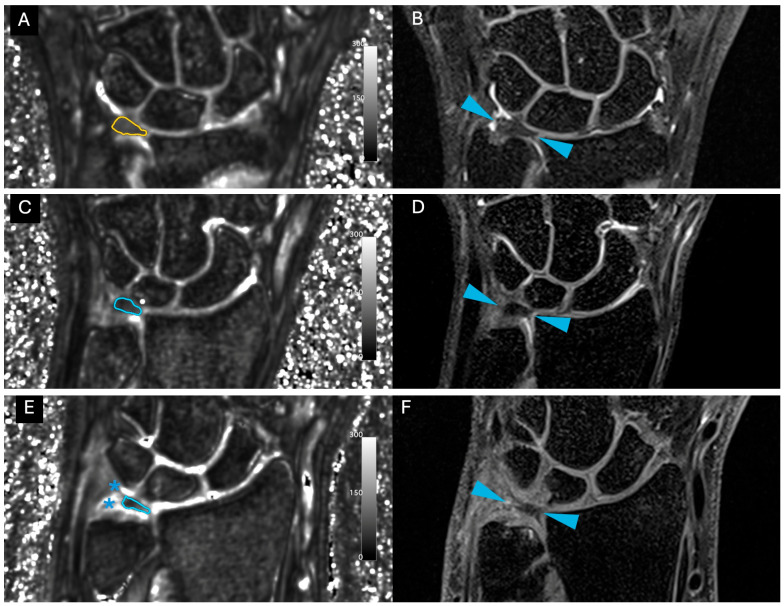
Visualization of a representative slice of the TFC disc for three different volunteers. (**A**,**C**,**E**): corresponding slice on the 3D UTE-T2* map with windowing parameters set between 0 and 300 a.u., corresponding to 0 and 30 ms. The manual contouring of the disc is shown in color. Stars indicate tendons that were excluded from the disc segmentation. (**B**,**D**,**F**): corresponding 3D PD SPACE with fat suppression in the coronal orientation. Fat suppression helps distinguish fluids from fat, as the latter is also hyperintense. Blue arrows indicate TFC. From top to bottom, the volunteers were 22, 33 and 48 years old, respectively.

Images were free of artefact, allowing optimal visualization of the TFCC in all cases. No cases of central TFC perforation or additional TFCC injuries were detected. An example of the sequences acquired for one volunteer is shown in Figure 1. Figure 2 presents the multiple echoes of the 3D UTE-T2* mapping sequence along with the resulting UTE-T2* map. TFC segmentation on a single slice of the UTE-T2* map is illustrated in Figure 3 for three volunteers with different ages.

The ages of the subjects ranged from 21 to 58 years, median 35 years, comprising four women and six men. All subjects were right-handed, with BMIs ranged between 21 and 29, mean was 24 ± 2.6 (considered as normal). The ROI volume of the TFC disc ranged from 0.105 and 0.268 cm^3^, and T2* relaxation times ranged from 6.7 to 13.9 ms. Details of epidemiologic data and T2* measurements are provided in Table 2.

**Figure 4:**
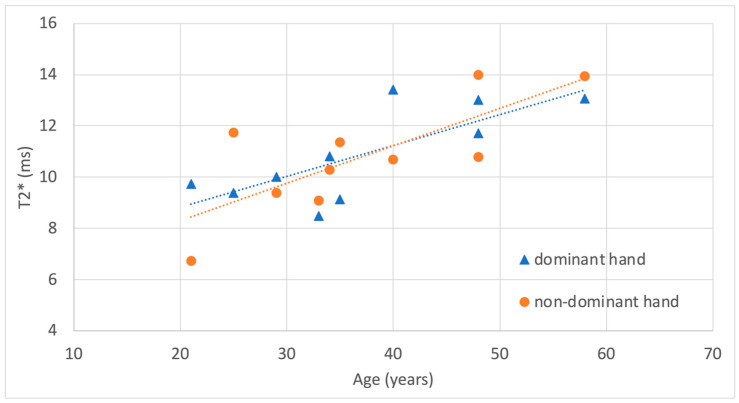
Correlation of T2* measurements with subject age, shown separately for dominant and non-dominant hands. Regression lines are included for visualization purposes.

**Figure 5:**
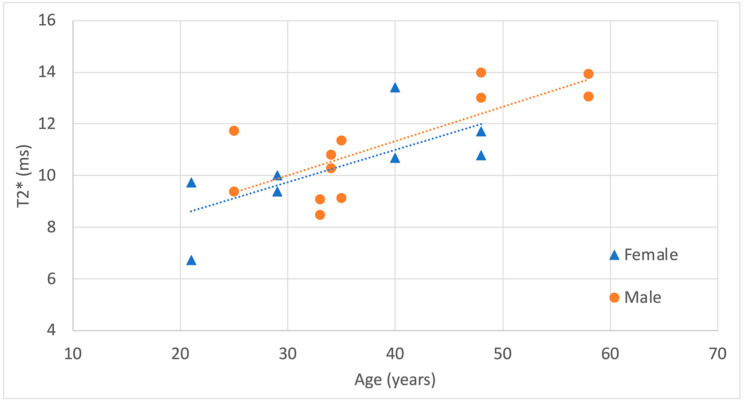
Correlation of T2* measurements with subject age, shown separately for males and females. Regression lines are included for visualization purposes.

The correlation of T2* values with age, sex, and dominant hand was analyzed using a linear regression model incorporating these three factors. T2* was positively correlated with age, R^2^ = 0.59, β-age = 0.13, CI 95% [0.070–0.19], *p* = 1.2×10^−4^. However, T2* was not significantly influenced by sex, β-sex = 0.32, CI 95% [−1.0–1.67], *p* = 0.61 or dominant hand, b-dominant hand = 0.07, CI 95% [−1.2–1.36], *p* = 0.91. Results are visualized separately for dominant and non-dominant hands, as well as for sex, in Figure 4 and Figure 5.

Interactions between age and sex, as well as between age and dominant hand, were tested using a more complex linear regression model. This model included the main factors of age, sex, and dominant hand, along with the interaction terms for age × sex and age × dominant hand, to assess potential moderating effects on T2* values. No significant interactions were found in our data, β-age:sex = 0.055, CI 95% [−0.13–0.14], *p* = 0.93 and β-age:dominant hand = −0.025, CI 95% [−0.15–0.10], *p* = 0.68. Additionally, there was no statistical difference between the simpler model (without interaction terms) and the more complex model (with interaction terms), *p* = 0.91.

Ulnar variance ranged from −3.16 to 0.55 mm. It was neutral in 12 cases and negative in 8 cases, with no positive ulnar variance measured. Within the limited range of ulnar variance of this cohort, T2* relaxation times were not correlated with ulnar variance, R^2^ = 0.033, β = −0.46, CI 95% [−1.7–0.76], *p* = 0.44.

## 4. Discussion

This pilot study revealed a successful application of UTE-T2* quantitative MRI for imaging the TFC in asymptomatic volunteers. Tissues were clearly visualized, and quantification of the TFC disc was feasible. As anticipated, T2* values of the TFC disc showed a strong correlation with subject age, while neither sex nor dominant hand had an impact on T2* values in our cohort.

Our findings are consistent with a previously published study examining TFC T2* measurements in different wrist positions [13]. In that study, subjects ranged in age from 27 to 35 years, and the UTE-T2* value for the prone wrist position was 7.92 ± 1.37 ms. In our cohort, the mean UTE-T2* value for subjects aged 27 to 35 years was 9.8 ± 0.98 ms. These results will help establish cutoff values for normal T2* relaxation times in the TFC, which are typically lacking and are essential for assessing pathological conditions.

Detection of compositional changes in fibrocartilage, a tissue with short T2* values, has been reported as a method to assess tissue deterioration prior to the appearance of morphological lesions. For instance, a correlation between UTE T2* relaxation time and degeneration has been observed in the meniscus. Nebelung et al. reported a strong positive correlation between histological scoring of meniscal degeneration and UTE T2* values, demonstrating high sensitivity for T2* measurements [22]. Additionally, they showed an overall increase in T2* relaxation time as meniscus degeneration progressed. Histologically, this change is attributed to a decrease in fibrocartilage cells and an increase in extracellular water, contrasting with healthy joints both in vitro and in vivo [26]. Furthermore, collagen, the second major component of the fibrocartilage extracellular matrix, is highly disturbed in degenerative processes, leading to less restricted water motion and an elevation of T2* values [27]. Similar findings have been reported in studies of the temporomandibular joint (TMJ), where symptomatic subjects showed higher T2* values compared to healthy volunteers, and T2* relaxation times were prolonged in degenerative discs [21]. Moreover, comparisons of histological and biomechanical characteristics of cadaveric TMJ discs with UTE-T2* measurements revealed an inverse relationship between collagen organization, stiffness, and T2* values [28]. These changes are related to alterations in water content, proteoglycans, and the collagen network during degeneration [29].

When comparing the meniscus or TMJ disc structure with the TFC histological composition, the TFC is predominantly composed of collagen, which is tightly packed at the center and along the radial side, contributing to its role as a shock absorber and stabilizer against multidirectional forces (such as pronation and supination). On the ulnar side of the TFC, fibers are oriented parallel and are more exposed to traction forces [30]. Immunohistochemical studies have shown that collagen in the TFC is exclusively type II, with glycosaminoglycans present in all regions of the disc and proteoglycans in the radial side—molecules that are characteristic of a cartilaginous phenotype [31]. Therefore, age-related alterations in these microstructures may explain the changes in quantitative MRI parameters observed in the TFC, as attested in our study.

Regarding positive ulnar variance, degenerative changes in the TFC are associated with wear of the central disc due to ulnar impaction. Ungualb et al. [4] demonstrated that positive ulnar variance promotes degeneration, and their subsequent studies confirmed that fibrochondrocyte apoptosis and cell loss occur in degenerative discs [3]. Biomechanical studies, both ex vivo and surgical, have shown that ulnar variance greater than 2 mm leads to concentrated pressure loading on the TFC [32]. In our study, no subjects with positive ulnar variance were observed, which limits the conclusions that can be drawn regarding this factor. However, additional research on this topic is needed, as ulnar variance remains a controversial factor in some studies, even though pronation has been shown to increase loading on the TFC [33]. Thus, we emphasize the only study conducted on UTE-T2* of the TFC that compares pronation and supination, confirming that acute loading during pronation leads to an increase in T2* values of the TFC [13].

Many studies have compared quantitative sequences such as T1, T1ρ, T2, T2* and UTE-T2*. UTE-T2* has shown high sensitivity to meniscus degeneration [22] and provides better delineation of meniscal tears or degeneration compared to T2* [20]. T2 is closely related to interstitial water content, which increases during degeneration [34], as well as to collagen organization [35]. UTE T2* appears to be more accurate for detecting cartilage or fibrocartilage (meniscal) tears or degeneration than T2*, as reported by Yi et al. likely because the effect size of UTE-T2* is greater than that of T2* [36]. Notably, UTE-T2* provides superior sensitivity and specificity for detecting changes in fibrocartilage compared to traditional T2*. In another study, comparing T2* and UTE-T2* for disc detecting degeneration, UTE-T2* was found to be more accurate in the early stage of the degeneration process [18]. Since UTE-T2* can probe components with very short T2, it is particularly effective for detecting reduction in both water content and proteoglycan content. Based on these findings, we justify using UTE-T2* measurements instead of T2* in our study, even though T2* of TFC disc is not so short, it remains within the same order of magnitude as those reported in previous studies.

This study has several limitations. First, we did not segment the central disc of the TFC into radial, central, and ulnar zones to calculate relaxation times separately and evaluate the differences between these areas. This would have helped assess whether the central region is more sensitive to early degeneration, based on its microstructural architecture. Despite the high resolution of the 3D UTE sequence, macroscopic delineation of these zones in the TFC remains challenging on MRI, and more advanced techniques may be necessary in the future. Second, this study was not complemented by histological comparisons, we believe that such additional work is essential to correlate the biomechanical characteristics of the central disc with quantitative imaging parameter. Third, T2* measurements are sensitive to field inhomogeneities as well as to the magic angle effect. This represents an inherent limitation of the method. Although we took care to position every subject consistently, similar to how a patient would be positioned in a clinical setting, this factor may still contribute to data variability [37]. Fourth, other confounding factors such as BMI or specific physical activity levels were not included in the analysis. However, none of our volunteers were obese, and we assumed a normal activity level, as their professional backgrounds did not involve either professional sports or manual labor. Finally, in the absence of histological comparison, a longitudinal study should be conducted to correlate T2 increases with disc degeneration due to aging.

Future studies should include patients with pathologies as well as pathological correlations to confirm the clinical utility of UTE-T2* as a relevant quantitative biomarker. Additionally, an inter-rater reliability study should be performed considering the manual segmentation process involved.

## 5. Conclusions

In conclusion, UTE-T2* showed positive correlation with subject age in TFC of healthy subjects. The higher T2* relaxation times may reflect early degeneration of the fibrocartilage microstructure, which is associated with both biomechanical factors and the aging process. These baseline values are a prerequisite for interpreting deviations observed in patients with suspected pathology in future studies.

## Figures and Tables

**Table 1:** Sequence parameters for the comprehensive wrist protocol.

Sequence	T1 TSE cor	PD TSE FS cor	PD TSE FS sag	3D PD SPACE FS cor	3D UTE FS cor	3D UTE-T2* Mapping
FOV (mm)	90	90	90	110	110	110
slice thickness (mm)	2.5	2.5	2.5	0.5	0.4	0.5
TE (ms)	13	37	37	44	0.04	0.04, 2.38, 5.62, 8.41
TR (ms)	505	2200	2200	900	5.7	13.7
Averages	2	2	2	1.4	1	1
flip angle (excitation)	90	90	90	PD Var §§	6	5
matrix	384	320	320	224	304	240
in-plane resolution (mm)	0.1	0.1	0.1	0.2	0.4	0.5
Deep Resolve §	Yes	Yes	Yes	No	No	No
Parallel imaging technique	GRAPPA	GRAPPA	GRAPPA	Compressed Sensing	none	none
Parallel imaging acceleration factor	3	2	2	5	-	-
Fat suppression	none	Fat saturation	Fat saturation	SPAIR	Fat saturation	none
Bandwidth (Hz/px)	303	252	252	413	715	906
Acquisition time (min:s)	02:32	02:00	02:00	04:18	03:51	05:57

^§^ Deep learning technology for image reconstruction- Siemens Healthineers; ^§§^ flip angle mode corresponding to PD contrast; Abbreviations: FOV field of view, TSE turbo spin echo, UTE ultra-short echo time, TE echo time, TR repetition time.

**Table 2:** Epidemiologic data and T2* measurement results for each subject.

Subject	Sex	Age (years)	Weight (kg)	Height (cm)	BMI	Side	ROI Volume (cm^3^)	Mean T2* (ms)	SD T2* (ms)	Dominant (D) or Non-Dominant (ND) Hand
1	M	34	83	180	26	Right	0.220	10.8	3.47	D
1	M	34	83	180	26	Left	0.105	10.3	2.65	ND
2	M	35	71	171	24	Right	0.188	9.1	3.14	D
2	M	35	71	171	24	Left	0.234	11.4	2.95	ND
3	M	33	57	170	23	Right	0.233	8.5	2.52	D
3	M	33	57	170	23	Left	0.255	9.1	2.32	ND
4	M	48	95	181	29	Right	0.121	13.0	6.64	D
4	M	48	95	181	29	Left	0.139	14.0	5.48	ND
5	F	21	58	168	21	Right	0.127	9.7	2.92	D
5	F	21	58	168	21	Left	0.175	6.7	2.24	ND
6	M	58	88	182	27	Right	0.188	13.1	5.63	D
6	M	58	88	182	27	Left	0.168	13.9	6.95	ND
7	F	29	57	165	21	Right	0.200	10.0	3.11	D
7	F	29	57	165	21	Left	0.210	9.4	2.53	ND
8	F	48	58	160	23	Right	0.115	11.7	3.68	D
8	F	48	58	160	23	Left	0.131	10.8	2.55	ND
9	F	40	62	169	22	Right	0.162	13.4	3.90	D
9	F	40	62	169	22	Left	0.131	10.7	3.64	ND
10	M	25	79	179	25	Right	0.268	9.4	3.60	D
10	M	25	79	179	25	Left	0.171	11.7	4.34	ND

## Data Availability

Data that are not published in the present article can be requested to the corresponding author.

## Abbreviations

The following abbreviations are used in this manuscript:

MRI	Magnetic Resonance Imaging
GRAPPA	GeneRalized Autocalibrating Partial Parallel Acquisition
PD	Proton Density
SPACE	Sampling Perfection with Application optimized Contrast using different flip angle Evolution
SPAIR	Spectral Attenuated Inversion Recover
TFC	Triangular Fibrocartilage
TFCC	Triangular Fibrocartilage Complex
UTE	Ultrashort echo time
